# Teeth with Exposed Pulps*It is a matter of regret that it has been impossible to find space for this article until now. It was sent in time for the December number, and the delay in answering the critic has not been the fault of the author.—Editor.

**Published:** 1887-04

**Authors:** B. Merrill Hopkinson


This is a matter, gentlemen, worthy of consideration, and not beneath the dignity of our calling.

What are we going to do about it?

### TEETH WITH EXPOSED PULPS.*

BY B. MERRILL HOPKINSON, D. D. S., M. D.

The author of the paper upon the above theme which appeared in the July No. of THE INDEPENDENT PRACTITIONER, having read the criticism upon it in the Oct. No., takes great pleasure in correcting the errors into which the critic appears to have fallen. The writer is glad that his paper has been thought worthy of examination, although he is surprised at some of the points taken by his opponent for discussion. He has nothing to add or retract. His *modus operandi* must stand as set forth in THE INDEPENDENT PRACTITIONER, and the record of many hundreds of pulpless teeth treated and filled, with no failures, is the strongest argument he can offer regarding the efficiency of the method in his hands.

The first point in the criticism is based upon a misapprehension of the pathological condition of the case mentioned by the author in his paper, and it is upon this premise that a large part of the article of the opposition is founded. From his remarks the reader would be led to infer that the author had not devitalized the living portion of a pulp, which pulp he admitted was partially dead, by speaking of the presence of pus, indicating, as the writer is well aware, decomposition after death. The author's critic says: "The indication to most of us would be that devitalization had already taken place in the body of the pulp." The presence of pus at the point of pulp exposure does not indicate, in many instances, more than the termination of a localized inflammation just at the point where the pulp is exposed, beyond which may be found irritated or inflamed, but still living tissue. The practice of the writer, as he has before stated, is to devitalize the living portion of such a pulp, and in so doing relieve his patient from distress, as well as hasten the death of the living part, which, "if you give it time," would unquestionably after a "few hours or days" or weeks of time, together with exquisite torture, likewise die from exposure. It is sincerely to be hoped that our brother in Chicago does not wait "a few hours or

*It is a matter of regret that it has been impossible to find space for this article until now. It was sent in time for the December number, and the delay in answering the critic has not been the fault of the author.—EDITOR.

days of time when a line of demarkation and practical separation will exist between such root filaments and the dental nerve," before he renders his patients such service as may relieve them from pain. The writer will add in this connection that experience teaches him that the vitality of the dental pulp is frequently most persistent, and he has known of many cases where such vitality has remained, when the pulp has been fully exposed, for not only a "few hours or days of time," but for weeks, when, for reasons best known to the individual, a preference is shown for continuous or intermittent pain, rather than to consult a specialist and obtain immediate relief.

There are many practitioners, advanced men, for whom we have the greatest respect and admiration, who pursue a very different course from that advocated by the writer with regard to saving exposed pulps, but all agree with him concerning the existence of vitality, in many cases, where suppuration is present at the point of exposure. Let us quote Dr. Wm. H. Atkinson, and, while the author has written in opposition to the precepts of this most able man, he can but respect them greatly, and when a suitable and convenient opportunity offers he will endeavor, if possible, to profit by them. Dr. Atkinson says (*vide* INDEPENDENT PRACTITIONER, May No. page 256): "If the pulp be sufficiently exposed we can very readily see if there is a drop of pus already formed at the point of exposure. If there is a drop of pus there, the character of that is almost certain to be a favorable indication for saving the pulp." He evidently does not think that devitalization has "taken place in the body of the pulp," and in this he represents a large class of scientific and practical men, many of whom could be quoted, if necessary, in support of this fact.

We may see very clearly, then, that a portion, and a large portion, of a pulp may still be, and frequently is, alive in a tooth, while immediately at the point of exposure decomposition and death of the tissue has taken place, as shown by an oozing of the pus. The pus oozes forth because it is under pressure, and by enlarging the point of exposure and relieving the pressure alleviation to the patient is sometimes afforded, but only very slight in degree when a complete exposure has existed from which pus has previously flown, and it is only after a suitable application has been made that the individual experiences complete relief. In some cases violent pain for a brief period follows the use of the drugs named in the author's paper, of itself evidence of remaining vitality, to be speedily followed by entire freedom from suffering. In those cases where there is only a partial exposure, viz., when a thin layer of dentine confines the pus, instant relief is afforded when that layer is removed and a free vent secured for the flow of pus. This, however, is only temporary, unless the entire contents of pulp chamber and root canals be already dead, and must be followed by appropriate treatment according to the views of each individual operator, in order to secure permanent immunity from distress. Having seen, then, that an exposed pulp, suppurating at the point of full exposure, is not necessarily a dead pulp, the reason "why was the patient in distress when he entered?" is shown to be the presence of suppurating, dead tissue, exercising pressure upon, in contact and in conjunction with, inflamed living tissue in the pulp chamber and root canals.

"Why is it relief is experienced by the individual in distress?" is the doctor's next interrogation, and we are told, "because a point of no resistance has been made by the excavator." The author thinks the "resistance" theory has been sufficiently explained away and requires no further comment, and that he has satisfactorily answered the above question. Cases in which suppuration of the pulp appears differ greatly, have different symptoms, and require different treatment. The intention of the passage criticised was simply to show the author's manner of devitalization as applied to all cases requiring such procedure, and for which there was no claim for originality.

As the "Echo" did not deign to give an answer to the question concerning the "principles of hydrostatics," the writer will also pass it by with the remark that a fluid (pus) will by its weight readily find a point of pulp exposure, if one exist.

The writer is always pleased to have his modes of operating criticised, for by genuine criticism and discussion new thoughts and ways are brought to light, and improvement is to be looked for in all who thus engage the mind; but he must add, in conclusion, that he does not regard the larger part of the paper to which this is answer, as one the reading of which would be instructive or beneficial, and he would respectfully recommend that future strictures upon others should be marked by more of candor and less of sarcasm and captiousness.

